# Oxygen gradient ektacytometry‐derived biomarkers are associated with vaso‐occlusive crises and correlate with treatment response in sickle cell disease

**DOI:** 10.1002/ajh.26031

**Published:** 2020-11-11

**Authors:** Minke A. E. Rab, Celeste K. Kanne, Jennifer Bos, Brigitte A. van Oirschot, Camille Boisson, Maite E. Houwing, Jorn Gerritsma, Erik Teske, Celine Renoux, Jurgen Riedl, Roger E. G. Schutgens, Marije Bartels, Erfan Nur, Philippe Joly, Romain Fort, Marjon H. Cnossen, Richard van Wijk, Philippe Connes, Eduard J. van Beers, Vivien A. Sheehan

**Affiliations:** ^1^ Central Diagnostic Laboratory‐Research University Medical Center Utrecht, Utrecht University Utrecht The Netherlands; ^2^ Van Creveldkliniek, University Medical Center Utrecht Utrecht University Utrecht The Netherlands; ^3^ Department of Pediatrics Emory University School of Medicine Childrenʼs Healthcare of Atlanta Atlanta Georgia USA; ^4^ Laboratory LIBM EA7424, University of Lyon 1, “Vascular Biology and Red Blood Cell” team Lyon France; ^5^ Laboratory of Excellence GR‐Ex Paris France; ^6^ Department of Pediatric Hematology Erasmus Medical Center Rotterdam The Netherlands; ^7^ Emma Childrenʼs Hospital, Pediatric Hematology Amsterdam University Medical Centers Amsterdam The Netherlands; ^8^ Department of Clinical Sciences of Companion Animals, Faculty of Veterinary Medicine Utrecht University Utrecht The Netherlands; ^9^ Laboratory of Biochemistry and Molecular Biology, UF Biochemistry of Red Blood Cell diseases, Est Center of Biology and Pathology Hospices Civils de Lyon Lyon France; ^10^ Result Laboratory Albert Schweitzer Hospital Dordrecht The Netherlands; ^11^ Department of Hematology Amsterdam University Medical Centers Amsterdam The Netherlands; ^12^ Department of Internal Medicine Hospices Civils de Lyon Lyon France

To the Editor:

Sickle cell disease (SCD) is an inherited hemoglobinopathy caused by a single nucleotide mutation in the β‐globin gene leading to the production of an abnormal hemoglobin S (HbS). When HbS becomes deoxygenated it polymerizes, deforming the red blood cells (RBCs) into a curved or sickle shape. Sickle RBCs are rigid and poorly deformable, and together with other abnormalities, block the microvasculature, causing painful vaso‐occlusive crises (VOC), the hallmark complication of SCD. Hemolysis, increased RBC adhesion, endothelial dysfunction, inflammation, oxidative stress, and hemostatic activation, also contribute to the development of VOC,^1^ although their relative contribution remains subject to debate. The non‐RBC factors are found in other disease conditions, yet no other disease is associated with VOC, highlighting the key contribution of sickled RBCs to SCD pathophysiology.^1^

Oxygen gradient ektacytometry is a newly developed method that characterizes sickling behavior.^2^ This functional assay measures deformability of the total RBC population over a range of oxygen concentrations, providing parameters that reflect features of sickling behavior and RBC function. The most important parameters are: (a) EI_max_, RBC deformability when RBCs are fully oxygenated; (b) EI_min_, lowest RBC deformability when HbS polymerization is at its peak value due to deoxygenation; and (c) point of sickling (PoS): the oxygen tension at which a 5% decrease in oxygenated EI_max_ is observed during the first minutes of deoxygenation, reflecting the patient‐specific pO_2_ at which HbS polymers are increasing and sickling of RBCs, that are able to deform under normoxic conditions, begins (Figure 1E).^2^

**FIGURE 1:**
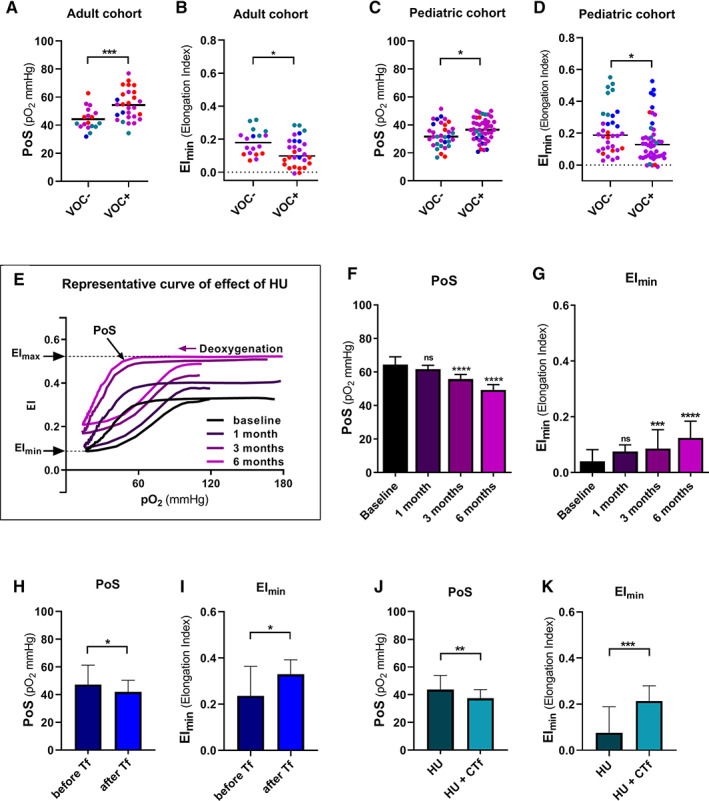
Oxygen gradient ektacytometry‐derived biomarkers are associated with VOC and improve with standard of care therapy. A, Point of Sickling (PoS) is significantly higher in patients with SCD in the adult cohort who experienced one or more VOC in the past 2 years (VOC+). Colors show different treatment regimens: untreated (red), HU treatment (purple), chronic transfusion (blue), HU and chronic transfusion (turquoise). B, Minimum deformability (EI_min_) is significantly higher in patients who did not experience a VOC in the past 2 years (VOC−) compared to patients who did, in the adult cohort. C, PoS is significantly higher in the VOC+ group in the pediatric cohort. D, EI_min_is significantly lower in the VOC+ group. The effect of starting HU therapy was measured in 15 patients with SCD at baseline, and after 1, 3 and 6 months of HU therapy. E, Representative curve of a patient before and during hydroxyurea (HU) titration to maximum tolerated dose. F, Median values of point of sickling (PoS) before and during HU therapy. PoS significantly decreases after 3 and 6 months compared to baseline values. G, Median values of minimum deformability upon deoxygenation (EI_min_) before and during HU therapy. EI_min_significantly increases after 3 and 6 months of HU therapy compared to baseline values. Seven patients with SCD were followed just before and after transfusion therapy. H, Median values of PoS are significantly decreased after transfusion, compared to before transfusion values. Median values of EI_min_I, are significantly increased by transfusion. Twenty‐one patients with SCD were followed during HU and HU with CTf therapy. J, Median values of PoS before and on CTf that significantly decreases during CTf. Median values of EI_min_K, are increased with CTf. Error bars represent interquartile range. ****P < .0001, ***P < .001, **P < .01, *P < .*05*

Clinically relevant biomarkers are needed to serve as surrogate endpoints to evaluate new US Food and Drug Administration (FDA) approved therapies for SCD in subsequent clinical trials. Crizanlizumab and L‐glutamine were approved based on subjective clinical endpoints of number of VOC or time to next VOC. No functional (sickling) assay was included in the trial design of L‐glutamine^3^ or crizanlizumab, and they did not change any conventional laboratory values.^4^ Functional RBC tests like oxygen gradient ektacytometry may provide quantitative, clinically relevant biomarkers to serve as surrogate endpoints for subsequent clinical trials and for patient care.

In this study, we initiated clinical validation studies of oxygen gradient ektacytometry‐derived biomarkers by investigating the relationship between these biomarkers and frequency of VOC. In addition, we evaluated the ability of these biomarkers to detect RBC changes with initiation of hydroxyurea (HU) or transfusion therapy.

A total of 126 patients were enrolled at participating sites in The Netherlands, France or the United States (US). Peripheral blood samples from patients with no VOC in the past 2 years (VOC‐group) were compared to patients who experienced one or more VOC (VOC+ group) in the past 2 years. The VOC was defined as an acute pain event attributed to SCD requiring hospital admission, emergency room evaluation or an unplanned visit to the outpatient clinic. The European cohort was comprised of adult patients (n = 46) and the United States cohort consisted of pediatric patients (n = 80) aged 3‐18 years with HbSS, HbSβ^o^ or HbSβ^+^. Characteristics of VOC+ and VOC‐ cohorts are shown in Table S1. Oxygen gradient ektacytometry was performed as described in detail elsewhere (supplemental methods).^2^ Overall, patient demographics and laboratory parameters were comparable between the two cohorts with differences regarding age, percentage of patients on HU treatment, percentage of patients on HU and chronic transfusion (CTf). In the adult cohort, PoS differed significantly between VOC‐ group (median 41.6 mm Hg) and VOC+ group (median 53.7 mm Hg, *P* = .001, Figure 1A). The same was observed in the pediatric cohort (*P* = .0495, Figure 1C), which indicates that RBCs of patients without VOC can tolerate lower oxygen tensions before sickling occurs. EI_min_ in both cohorts was significantly lower in the VOC+ group (adult cohort *P* = .0178, pediatric cohort *P* = .022, Figure 1B,D), which highlights the fact that RBCs of patients in the VOC+ group are less deformable after deoxygenation. The EI_max_ was not significantly different between the VOC‐ and VOC+ groups in the pediatric cohort, but was significantly higher in the VOC‐ group in the adult cohort (Figure S1A,B).

To assess the association of oxygen gradient ektacytometry‐derived biomarkers with RBC characteristics and known indicators of disease severity, such as HbF and HbS levels, and markers of hemolysis (Hb, lactate dehydrogenase (LDH), bilirubin and absolute reticulocyte count(ARC), the degree of correlation between these laboratory parameters and PoS, EI_max_, EI_min_, were calculated for each cohort. We found significant correlations between EI_max_, PoS and EI_min_ with HbS and HbF levels. Various biomarkers correlated with ARC, bilirubin, LDH, ferritin, or creatinine in both cohorts (Tables S2 and S3).

To assess if oxygen gradient ektacytometry‐derived biomarkers changed in patients receiving or initiating standard of care treatment of transfusion or HU, we analyzed three patient cohorts on different treatment regimens. In the first cohort, 15 SCD patients were followed before and during HU treatment. After 3 and 6 months on HU the PoS decreased significantly and EI_max_ and EI_min_ increased accordingly (Figure 1E‐G; Figure S2; Table S4). Transfusion immediately improved EI_max_, PoS, and EI_min_, (all *P* < .05, Figure 1H,I, Figure S3, Table S5). In the cohort treated with CTf, PoS, EI_max_ and EI_min_ improved significantly (all *P* < .01, Figure 1J,K, Figure S4, Table S5). Importantly, while oxygen gradient ektacytometry‐derived biomarkers showed a considerable and significant change, most conventional laboratory tests did not (Table S5), confirming that this technique provides an additional insight into the efficacy of disease modifying therapies.

In our study, specific oxygen gradient ektacytometry‐derived biomarkers were associated with VOC in both cohorts, in contrast to conventional clinical laboratory parameters such as HbS and HbF levels and ARC. The PoS and EI_min_ were found to be the most reproducible clinically relevant biomarkers because they were associated with VOC in both adult and pediatric cohorts. The reproducible association observed, is likely due to the fact that oxygen gradient ektacytometry is a functional test that captures the combined effect of many factors contributing to RBC function, some measurable by clinical testing, like HbF and HbS levels, and some not, like the impact of 2,3‐diphosphoglycerate levels, and oxidative stress.^2^

However, we observed differences in oxygen gradient ektacytometry‐derived biomarkers between cohorts. This could be due to differences in pediatric and adult SCD pathophysiology, in particular RBC rheological characteristics, and notably RBC deformability, which has been found to be different between adults and young children.^5^ In addition, other factors may also play a role, such as (a) differences in access to care (US vs Europe), (b) differences in guidelines, clinical practice and health care between adult and pediatric patients, (c) cultural differences between sites, particularly response to pain, (d) environmental factors such as climate, or (e) technical differences.

Limitations of oxygen gradient ektacytometry‐derived biomarkers are that they primarily evaluate RBC function, and do not capture other factors important in SCD pathophysiology. Inclusion of other factors, such as soluble vascular cell adhesion molecule, or other functional tests, like adhesion to an artificial microfluidic network could complement this technology.

There are four FDA approved pharmacologic therapies available for routine SCD care in the US. The best way to choose which drug to prescribe to which patients is a topic of intense discussion. There are also several other agents currently in clinical trials in the US and in Europe. Clinically relevant biomarkers, such as oxygen gradient ektacytometry, are needed for several indications: (a) to select the optimal therapy based on the patientʼs particular blood rheology, according to the principles of precision medicine, (b) to monitor efficacy in an objective, rapid manner rather than watching for clinical complications which can take years to emerge, and (c) to provide objective clinically relevant endpoints for clinical trials. Biomarkers of RBC function, like those provided by oxygen gradient ektacytometry, could also be excellent ways to assess gene‐based therapy outcomes. The amount of HbF induction needed to be achieved by gene therapy, or amount of HbS reduction by allogeneic stem cell transplantation to affect a cure is still a topic for discussion. In most clinical trials sickling behavior is not functionally assessed, as microscopy based sickling assays are static and do not provide information on patient specific sickling characteristics.^6^ Additionally, different gene therapy trials induce different functional hemoglobins, and a functional analysis of the resulting RBC population is the best way to compare different strategies head to head. The VOC is still the primary endpoint in ongoing gene therapy trials, however lack of VOC does not demonstrate a cure as organ damage may still be ongoing. Functional biomarkers like EI_min_ and PoS could better assess the effectivity of these therapies in individual patients, indicating if the treatment was successful enough to prevent clinically relevant sickling.^7^

In conclusion, oxygen gradient ektacytometry‐derived biomarkers provides functional, clinically relevant next generation biomarkers that are associated with VOC. This can provide the clinician with information about patient RBC characteristics and sickling propensity that could eventually aid in clinical decision making. Moreover, oxygen gradient ektacytometry captures several RBC characteristics that have additional value over conventional laboratory tests. We have shown that oxygen gradient ektacytometry‐derived biomarkers improve with known efficacious therapies and are associated with VOC; therefore, these biomarkers can be an objective surrogate endpoint of disease modification.

## CONFLICT OF INTEREST

M.A.E.R., J.B., and B.A.vO. received grant funding from RR Mechatronics. R.v.W. received grant funding from Agios Pharmaceuticals Inc and RR Mechatronics. E.J.v.B. received grant funding Agios Pharmaceuticals Inc, RR Mechatronics, Novartis and Pfizer for investigator initiated research projects. V.A.S. received grant funding from Global Blood Therapeutics, Emmaus, and Novartis for investigator initiated research projects.

## AUTHOR CONTRIBUTIONS

Contribution: Minke A.E. Rab, Richard van Wijk, Eduard J. van Beers, and Vivien A. Sheehan designed the study; Minke A.E. Rab, Maite E. Houwing, Jorn Gerritsma, Celine Renoux, Philippe Joly, Marije Bartels, Erfan Nur, Romain Fort, Maite E. Houwing, Eduard J. van Beers, and Vivien A. Sheehan collected clinical and laboratory data. Minke A.E. Rab, Celeste K. Kanne, Jennifer Bos, Brigitte A. van Oirschot, Erik Teske and Jurgen Riedl and Camille Boisson performed laboratory experiments. Minke A.E. Rab, Richard van Wijk, Philippe Connes, Eduard J. van Beers, and Vivien A. Sheehan analyzed the data and wrote the manuscript. All authors edited the manuscript and approved the final version.

## Supporting information


**Appendix S1**. Supplemental methods.

**Table S1**. Characteristics of SCD patients with and without a history of VOC.
**Table S2**. Correlations of oxygen gradient ektacytometry‐derived biomarkers with laboratory parameters in the adult VOC‐/VOC+ cohort.
**Table S3**. Correlations of oxygen gradient ektacytometry derived‐biomarkers with laboratory parameters in the pediatric VOC‐/VOC+ cohort.
**Table S4**. Effect of HU on oxygen gradient ektacytometry‐derived biomarkers and laboratory parameters.
**Table S5**. Effect of a single transfusion or chronic transfusion therapy on oxygen gradient ektacytometry‐derived biomarkers and laboratory parameters.
**Figure S1**. Oxygen gradient ektacytometry‐derived biomarkers are associated with vaso‐occlusive crisis. (A) Maximum deformability (EI_max_) is significantly lower in patients in the adult cohort with VOC compared to those without. Colors show different treatment regimens: untreated (red), HU treatment (purple), chronic transfusion (blue), HU and chronic transfusion (turquoise). (B) EI_max_ is not significantly different in patients in the pediatric cohort with VOC compared to those without. **P < .01.
**Figure S2**. Hydroxyurea has a measurable effect on oxygen gradient ektacytometry‐derived biomarkers. The effect of starting HU therapy was measured in 15 patients with SCD at baseline, and after 1, 3 and 6 months of HU therapy. (A) Representative curve of a patient before and during hydroxyurea (HU) titration to maximum tolerated dose. (B) Median values of maximum deformability before deoxygenation (EI_max_) just before and during HU therapy. EI_max_ significantly increases after 3 and 6 months of HU compared to baseline values. Error bars represent interquartile range. ****P < .0001, ***P < .001, **P < .01, *P < .*05*.
**Figure S3**. A single blood transfusion has a measurable effect on oxygen gradient ektacytometry‐derived biomarkers. Seven patients with SCD were followed just before and after transfusion therapy. (A) Representative curve that highlights how blood rheology is improved by a blood transfusion. (B) Median values of EI_max_ are significantly decreased after transfusion, compared to before transfusion values. Error bars represent interquartile range. *P < .*05*.
**Figure S4**. Chronic transfusion improves oxygen gradient ektacytometry‐derived biomarkers in pediatric patients already treated with hydroxyurea therapy. Twenty‐one patients with SCD were followed during HU and HU with CTf therapy. (A) Representative curve of a patient on hydroxyurea (HU) therapy before start of chronic transfusion therapy (CTf) and on CTf. (B) Median values of EI_max_ before and on CTf, that significantly decreases during CTf. Error bars represent interquartile range. **P < .*01*.

## Data Availability

The data that supports the findings of this study are available in the supplementary material of this article. Additional data that support the findings of this study are available from the corresponding author upon reasonable request.
