# Supplementary material for: Oxygen gradient ektacytometry‐derived biomarkers are associated with vaso‐occlusive crises and correlate with treatment response in sickle cell disease
Source: Am J Hematol. 2020 Nov 11;96(1):E29–32. doi: 10.1002/ajh.26031 (PMC7756395; doi:10.1002/ajh.26031)
Supplement: Supplementary file 1 — Appendix S1. Supplemental methods. [file AJH-96-E29-s001.docx]

**SUPPLEMENTAL METHODS**

**Patients**A total of 126 patients were enrolled at participating sites in The Netherlands (University Medical Center Utrecht, Erasmus Medical Center Rotterdam, Amsterdam University Medical Centers), in France (Department of Internal Medicine and Institute of Pediatric Hematology and Oncology, Hospices Civils de Lyon) or the United States (Texas Children’s Hematology Center (TCHC)). Study procedures, namely collection of an additional tube of peripheral blood and review of medical records, were approved by local ethical committee/internal review boards in accordance with the Declaration of Helsinki, under registration number 17/392, 17/450, and H35473 at Baylor College of Medicine. All patients or legal guardians gave informed consent.

Peripheral blood samples from patients with no VOC in the past two years (VOC- group) were compared to patients who experienced one or more VOC (VOC+ group) in the past two years. VOC was defined as an acute pain event attributed to SCD requiring hospital admission, emergency room evaluation or an unplanned visit to the outpatient clinic. The European cohort was comprised of adult patients (n=46) with homozygous HbS (HbSS), HbS/β^o^-thalassemia (HbSβ^o^) or HbS/β^+^-thalassemia (HbSβ^+^). The United States cohort consisted of pediatric patients (n=80) aged 3-18 years with HbSS, HbSβ^o^ or HbSβ^+^. Pediatric patients under three years of age were excluded because of low incidence of VOC in this age group.

To assess the effect of established SCD treatments on oxygen gradient ektacytometry biomarkers, a cohort of patients were assessed before and during the first 6 months of HU therapy (n=15). Patients who received transfusion were excluded from this cohort. A second cohort was assessed before and during transfusion therapy, with variable time between blood collection and last transfusion (median 27; range 5-77 days; n=21). Additionally, in a cohort of patients receiving regular monthly transfusions, the immediate effect of a single transfusion was assessed (n=7).

**Laboratory tests**Laboratory tests, including complete blood count, absolute reticulocyte count (ARC), and hemoglobin profile, were obtained as part of routine visits to the outpatient clinic prior to oxygen gradient ektacytometry measurements. Percentage dense RBCs (%DRBCs, defined as RBC with a hemoglobin concentration of 41 g/dL or higher) were measured with an ADVIA 120/2120 hematology analyzer (Siemens, Healthcare GmBH, Erlangen, Germany).

**Oxygen gradient ektacytometry**Lyon or UMC Utrecht samples were measured by oxygen gradient ektacytometry after being stored at 4^O^C for 24hours TCHC samples were measured 8 hours after collection. Oxygen gradient ektacytometry was carried out with the Laser Optical Rotational Red Cell Analyzer (Lorrca, RR Mechatronics, Zwaag, The Netherlands). The oxygenscan is a new add-on feature of the Lorrca which allows measurement of red cell deformability as a function of continuously changing oxygen tensions, described in detail elsewhere.^1–3^ Briefly, to carry out the oxygen gradient ektacytometry, 50 µL of whole blood, standardized to a fixed RBC count of 200x10^6^, is suspended in 5 mL Oxy-Iso (RR Mechatronics, osmolarity 282-286 mOsm/kg, pH 7.35-7.45 at room temperature 21± 1^O^C).^2,3^

**Statistical Analysis**
A curve fit was calculated for every individual measurement with R studio based on Pearson’s correlation, generating the additional oxygen gradient ektacytometry-derived biomarkers slope and EI_0_ (Figure 1). EI_0_ indicates the calculated RBC deformation when oxygen tension is lowered to 0.0 mmHg. Slope reflects the rapidity of RBC sickling during deoxygenation (Figure 1).
Mann-Whitney test was performed to compare values from the VOC+ and VOC- groups and a Wilcoxon signed-rank test to compare values pre- and on therapy. All correlations were done with univariate Spearman’s correlation. GraphPad Prism (version 8.0) was used in all statistical analysis except for curve fit analysis. A *p-value* <0.05 was considered statistically significant.

**SUPPLEMENTAL RESULTS**

**Oxygen gradient ektacytometry-derived biomarkers are associated with vaso-occlusive crisis frequency**Characteristics of VOC+ and VOC- cohorts are shown in Supplemental Table 1. Overall, patient demographics and laboratory parameters were comparable between the two cohorts. Differences were noted with regard to age (significantly higher in the VOC- group of the adult cohort), percentage of patients on HU treatment (higher in the VOC+ group in the pediatric cohort), percentage of patients on HU and chronic transfusion (CTf) (higher in the VOC- group in both cohorts). Laboratory parameters were not significantly different in the pediatric cohort, but in the adult patient cohort DRBCs and bilirubin were significantly higher in the VOC+ group,

In the adult cohort, PoS differed significantly between VOC- group (median 41.6mmHg) and VOC+ group (median 53.7 mmHg, p=0.0008, Figure 2A). The same was observed in the pediatric cohort (p=0.0495, Figure 2D), which indicates that RBCs of patients without VOC can tolerate lower oxygen tensions before sickling occurs. EI_min_ in both cohorts was significantly lower in patients who experienced VOC (adult cohort p=0.0178, pediatric cohort p=0.022, Figure 2C and F), which highlights the fact that RBCs of patients in the VOC+ group are less deformable at the end of the deoxygenation period.

EI_max_ was not significantly different between the VOC- and VOC+ groups in the pediatric cohort, but was significantly higher in the VOC- group in the adult cohort (Figure 2B and E), indicating that at fully oxygenated conditions RBC deformability is lower in the VOC+ group compared to the VOC- group in adult SCD patients. Slope, indicating the rapidity of RBC sickling during deoxygenation, was significantly higher in in the adult VOC- group (p=0.0002) but not significantly different in the pediatric cohort (Supplemental Figure 1A and D). Higher Slope values indicate a homogenous population of RBCs that sickle at a low oxygen tension, which is favorable for the patient. Lower Slope values indicate a heterogeneous population consisting of a percentage of sickled cells at normoxia and a population of RBCs that start to sickle at a high oxygen tension, which is unfavorable. Oxygen gradient ektacytometry-derived biomarkers are population based; therefore, these findings could indicate that in the adult cohort RBCs of patients without VOC are composed of a more homogenous distribution of HbS than the pediatric cohort.

To assess the association of oxygen gradient ektacytometry-derived biomarkers with RBC characteristics and known indicators of disease severity, such as HbF and HbS levels, and markers of hemolysis (Hb, lactate dehydrogenase (LDH), bilirubin and ARC), the degree of correlation between these lab parameters and the biomarkers PoS, EI_max_, EI_min_, Slope, EI_0_ and DeltaEI were calculated for each cohort (Supplemental Table 1 and 2). In addition, markers of inflammation and organ damage such as ferritin and creatinine were assessed for degree of correlation with oxygen gradient ektacytometry-derived biomarkers as well. We found significant correlations between EI_max_, PoS and EI_min_ with HbS and HbF levels. Various biomarkers correlated with ARC, bilirubin, LDH, ferritin, or creatinine in the adult cohort (Supplemental Table 1). In the pediatric cohort comparable results were found, with the strongest correlation found between EI_max_ and %HbS (r= -0.895, p=0.001; Supplemental Table 2).

**Oxygen gradient ektacytometry-derived biomarkers are modulated by standard of care therapy**To assess if oxygen gradient ektacytometry-derived biomarkers changed in patients receiving or initiating standard of care treatment of transfusion or HU, we analyzed three patient cohorts on different treatment regimens. In the first cohort, 15 SCD patients (median age 13.0y, range 1.8-26.0, 7 female), were followed before and during HU treatment. Measurements were performed at baseline, and 1, 3 and 6 months after starting HU. Based on weight, patients were started 1000mg or 1500mg daily and titrated up by 500mg until maximum tolerated dose was achieved. During HU titration, oxygen gradient ektacytometry curves showed significant changes in most biomarkers (Figure 3A). After 3 and 6 months on HU the PoS decreased significantly from 64.4 to 55.8 and 50.7 mmHg, respectively (Figure 3B; Supplemental Table 3). Accordingly, EI_max_, EI_min_ and Slope increased significantly after 3 and 6 months of HU treatment (Figure 3C-E; Supplemental Table 3).

To assess the immediate effect of red cell transfusion, samples from seven SCD patients (included in The Netherlands, median age 26.2y, range 6.5-62.0; 6 female) obtained before and immediately after transfusion with 1-2 RBC units (6 patients received 2 units, 1 patient 1 unit) were analyzed by oxygen gradient ektacytometry. Transfusion immediately improved EI_max_, PoS, EI_min_, and EI_0_ (all p<0.05, Figure 4, Supplemental Table 4).

We assessed the effect of chronic transfusion on biomarkers in combination with HU treatment in a subgroup of 21 patients receiving care at TCHC (median age 7.6y; range 2.1-20.9, 6 female). PoS, EI_max_ and EI_0_ improved significantly (all p<0.01, Figure 5A-C and E). EI_min_ displayed the largest improvement (0.08 to 0.21 EI or 24.6% increase, p<0.001, Figure 5D). Importantly, while the oxygen gradient ektacytometry-derived biomarkers showed a considerable and significant change, most conventional laboratory tests did not (Supplemental Table 4), confirming that this technique provides an additional insight into the efficacy of disease modifying therapies.

**REFERENCES**

1. Rab MAE, van Oirschot BA, Bos J, Merkx TH, van Wesel ACW, Abdulmalik O, et al. Rapid and reproducible characterization of sickling during automated deoxygenation in sickle cell disease patients. Am J Hematol. 2019;94(February):575–84.

2. Rab MAE, van Oirschot BA, Bos J, Kanne CK, Sheehan VA, van Beers EJ, et al. Characterization of Sickling During Controlled Automated Deoxygenation with Oxygen Gradient Ektacytometry. J Vis Exp. 2019;(153):1–10.

3. Rab MAE, Kanne CK, Bos J, Boisson C, van Oirschot BA, Nader E, et al. Methodological aspects of the oxygenscan in sickle cell disease: A need for standardization. Am J Hematol. 2020;95(1):5–8.
