# Supplementary material for: Oxygen gradient ektacytometry‐derived biomarkers are associated with vaso‐occlusive crises and correlate with treatment response in sickle cell disease
Source: Am J Hematol. 2020 Nov 11;96(1):E29–32. doi: 10.1002/ajh.26031 (PMC7756395; doi:10.1002/ajh.26031)
Supplement: Supplementary file 2 — Table S1. Characteristics of SCD patients with and without a history of VOC. Table S2. Correlations of oxygen gradient ektacytometry‐derived biomarkers with laboratory parameters in the adult VOC‐/VOC+ cohort. Table S3. Correlations of oxygen gradient ektacytometry derived‐biomarkers with laboratory parameters in the pediatric VOC‐/VOC+ cohort. Table S4. Effect of HU on oxygen gradient ektacytometry‐derived biomarkers and laboratory parameters. Table S5. Effect of a single transfusion or chronic transfusion therapy on oxygen gradient ektacytometry‐derived biomarkers and laboratory parameters. Figure S1. Oxygen gradient ektacytometry‐derived biomarkers are associated with vaso‐occlusive crisis. (A) Maximum deformability (EImax) is significantly lower in patients in the adult cohort with VOC compared to those without. Colors show different treatment regimens: untreated (red), HU treatment (purple), chronic transfusion (blue), HU and chronic transfusion (turquoise). (B) EImax is not significantly different in patients in the pediatric cohort with VOC compared to those without. **P < .01. Figure S2. Hydroxyurea has a measurable effect on oxygen gradient ektacytometry‐derived biomarkers. The effect of starting HU therapy was measured in 15 patients with SCD at baseline, and after 1, 3 and 6 months of HU therapy. (A) Representative curve of a patient before and during hydroxyurea (HU) titration to maximum tolerated dose. (B) Median values of maximum deformability before deoxygenation (EImax) just before and during HU therapy. EImax significantly increases after 3 and 6 months of HU compared to baseline values. Error bars represent interquartile range. ****P < .0001, ***P < .001, **P < .01, *P < .05. Figure S3. A single blood transfusion has a measurable effect on oxygen gradient ektacytometry‐derived biomarkers. Seven patients with SCD were followed just before and after transfusion therapy. (A) Representative curve that highlights how blood rheology is improved by a bloo [file AJH-96-E29-s002.docx]

| **Supplemental Table 1.** **Characteristics of SCD patients with and without a history of VOC** | | | | |
| --- | --- | --- | --- | --- |
|  | **Adult cohort** | | **Pediatric cohort** | |
|  | **VOC–**  (n=18) | **VOC+**  (n=28) | **VOC-**  (n=34) | **VOC+**  (n=46) |
| **Patient related** |  |  |  |  |
| Sex, female n (%) | 11 (61) | 13 (46) | 15 (44) | 20 (43) |
| Age, years, median (range) | 41 (19-62) | 24 (18-45)**^§^** | 8 (3-18) | 12 (4-17) |
| HbS/β^+^-thalassemia, n (%) | 1 (6) | 0 (0) | 2 (6) | 1 (2) |
| α-Thalassemia: 1 deletion, n (%) | 4 (22) | 4 (14) | *nd* | *nd* |
| 2 deletions, n (%) | 1 (6) | 2 (7) | *nd* | *nd* |
| Splenectomy, n (%) | 5 (28) | 3 (11) | 9 (26) | 14 (30) |
| **Current treatment** |  |  |  |  |
| HU, n (%) | 8 (44) | 11 (39) | 18 (53) | 34 (74) |
| Dose HU, fixed or mg/kg, median (range) | 1000 (500-1500) | 1500 (500-2000) | 24 (12-35) | 26 (12-36) |
| Duration of HU, years, median (range) | 7 (4-10) | 3 (1-17) | 3 (1-11) | 5 (1-10) |
| Chronic transfusion, n (%) | 1 (6) | 3 (11) | 5 (15) | 6 (13) |
| HU + Chronic transfusion, n (%) | 5 (28) | 3 (11) | 7 (21) | 5 (11) |
| **Red cell characteristics** |  |  |  |  |
| HbF (%)^a^ | 15 ±11 | 9 ±9 | 9 ±10 | 9 ±8 |
| HbS (%)^a^ | 65 ±16 | 73 ±18 | 50 ±25 | 60 ±28 |
| Hb (g/dL) | 9.3 ±1.3 | 9.4 ±1.1 | 9.0 ±1.3 | 9.0 ±1.3 |
| ARC (10^9^/L)^b^ | 305 ±196 | 262 ±120 | 423 ±215 | 421 ±155 |
| MCV (fl) | 92 ±20 | 89 ±14 | 90±11 | 94±13 |
| MCHC (g/dL) | 34 ±2 | 35±1 | 32 ±1 | 33 ±1 |
| Dense RBCs (%)^c^ | 2.2 ±1.5 | 5.3 ±3.1***** | 4.0 ±2.1 | 5.0 ±3.6 |
| **Other laboratory characteristics** |  |  |  |  |
| Platelet count (10^9^/L) | 367 ±156 | 380 ±181 | 399 ±158 | 462 ±243 |
| Leucocytes (10^9^/L) | 8.8 ±2.6 | 9.4 ±3.6 | 9.7 ±3.4 | 10.5 ±4.7 |
| Neutrophils (10^9^/L) | 4.7 ±2.0 | 5.3 ±2.8 | 5.0 ±2.3 | 4.8 ±2.4 |
| Bilirubin (total (mg/dL) ^d^ | 0.32±0.15 | 0.58 ±0.32**†** | 2.7 ±1.8 | 2.5 ±0.9 |
| LDH (U/L) ^e^ | 362 ±129 | 463 ±164 | 1197 ±384 | 1305 ±641 |
| Ferritin (ng/mL) ^f^ | 1035 ±2151 | 300 ±349 | 1345 ±555 | 1913 ±686 |
| Creatinine (mg/dL) ^g^ | 0.71 ±0.23 | 0.65 ±0.17 | 0.44 ±0.14 | 0.37 ±0.09 |
| **Supplemental Table 1. Characteristics of the adult patient cohort and pediatric patient cohort in which patients who experienced VOC were compared to patients who did not.** Patients with no history of previous VOC in the past two years VOC-) are compared to patients who were admitted or assessed in the emergency room because of VOC in the past two years (VOC+). Numbers represent mean ± SDs or state otherwise. VOC, vaso occlusive crisis; HbS, hemoglobin S; HU, hydroxyurea, HbF, fetal hemoglobin; Hb, hemoglobin; ARC, absolute reticulocyte count, RBC, red blood cell; MCV, mean corpuscular volume; MCHC, mean corpuscular hemoglobin concentration; LDH, lactate dehydrogenase; nd, not determined; §p<0.001, †p<0.01, *p<0.05. | | | | |

^a^ In the pediatric cohort , data of 28 patients available, 13 in VOC- group, 15 in VOC+ group; ^b^ In the adult cohort, data of 42 patients available, 15 in VOC- group, 27 in VOC+ group; ^c^ In the adult cohort, data of 18 patients available, 6 in VOC- group, 12 in VOC+ group; ^d^ In the adult cohort, data of 40 patients available, 14 in VOC- group, 26 in VOC+ group. In the pediatric cohort, data of 33 patients available, 13 in VOC- group, 20 in VOC+ group; ^e^ In the adult cohort, data of 39 patients available, 12 in VOC- group, 27 in VOC+ group. In the pediatric cohort , data of 22 patients available, 7 in VOC- group, 15 in VOC+ group; ^f^ In the adult cohort, data of 41 patients available, 14 in VOC- group, 27 in VOC+ group. In the pediatric cohort , data of 15 patients available, 9 in VOC- group, 6 in VOC+ group; ^g^ In the adult cohort, data of 42 patients available, 15 in VOC- group, 27 in VOC+ group. In the pediatric cohort, data of 49 patients available, 19 in VOC- group, 30 in VOC+ group.

**Supplemental Table 2. Correlations of oxygen gradient ektacytometry-derived biomarkers with laboratory parameters in the adult VOC-/VOC+ cohort**

|  | **Adult cohort^a^** | | | |
| --- | --- | --- | --- | --- |
|  | **Oxygen gradient ektacytometry-derived biomarkers** | | | |
| **Laboratory parameter** | **EI_max_** | **PoS** | **EI_min_** |  |
| HbF (%) | **0.666^‡^** | **-0.461^†^** | **-0.499^†^** |  |
| HbS (%) | **-0.667****^‡^** | **0.399***** | **0.650^‡^** |  |
| Hb (g/dL) | **0.391*** | -0.198 | 0.276 |  |
| ARC (10^9^/L)^b^ | **-0.643^§^** | **0.421*** | -0.330 |  |
| MCV (fl) | 0.279 | -0.032 | 0.320 |  |
| MCHC (g/dL) | 0.071 | -0.087 | **0.520^†^** |  |
| Dense Cells (%)^c^ | **-0.950^‡^** | **0.736^†^** | -0.447 |  |
| Bilirubin (total (mg/dL)^d^ | **-0.593^§^** | **0.510^†^** | **-0.446*** |  |
| LDH (U/L)^e^ | **-0.484^†^** | 0.329 | -0.328 |  |
| Ferritin (ng/mL)^f^ | **0.368*** | -0.232 | **0.410*** |  |
| Creatinine (mg/dL)^g^ | -0.132 | 0.224 | -0.213 |  |

Supplemental Table 2. Correlations of oxygen gradient ektacytometry derived-biomarkers with laboratory parameters in the adult cohort.
HbF, fetal haemoglobin; HbS, haemoglobin S; Hb, haemoglobin; ARC, absolute reticulocyte count; MCV, mean corpuscular volume; MCHC, mean corpuscular haemoglobin concentration; LDH, lactate dehydrogenase; TSAT, transferrin saturation;
‡p<0.0001, §p<0.001, †p<0.01, *p<0.05.
^a^Total population of this cohort was 35 adult patients with SCA who were untreated or treated with hydroxyurea. Patients on (chronic) transfusion therapy (n=11) were excluded from this analysis.
^b^Data of 32 patients available, 3 patients had unknown data
^c^Data of 13 patients available, 22 patients had unknown data
^d^Data of 19 patients available, 16 patients had unknown data
^e^Data of 33 patients available, 2 patients had unknown data
^f^Data of 31 patients available, 4 patients had unknown data
^g^Data of 19 patients available, 16 patients had unknown data

**Supplemental Table 3.** **Correlations of oxygen gradient ektacytometry derived-biomarkers with laboratory parameters in the pediatric VOC-/VOC+ cohort**

|  | **Pediatric cohort^a^** | | | |
| --- | --- | --- | --- | --- |
|  | **Oxygen gradient ektacytometry-derived biomarkers** | | | |
| **Laboratory parameter** | **EI_max_** | **PoS** | **EI_min_** | |
| HbF (%)^b^ | **0.699*** | -0.397 | | **0.825^†^** |
| HbS (%)^b^ | **-0.895^§^** | **0.811^†^** | | **0.853^§^** |
| Hb (g/dL) | **0.631^‡^** | **-0.365^†^** | | **0.377^†^** |
| ARC (10^9^/L) | **-0.462^§^** | **0.272*** | | **-0.269*** |
| MCV (fl) | -0.004 | 0.136 | | 0.041 |
| MCHC (g/dL) | 0.042 | 0.113 | | 0.048 |
| Dense Cells (%) | **-0.768^‡^** | **0.668^‡^** | | **-0.572^‡^** |
| Bilirubin (total) (mg/dL)^c^ | **-0.464*** | **0.405*** | | -0.342 |
| LDH (U/L)^d^ | -0.296 | 0.122 | | 0.058 |
| Ferritin (ng/mL) | *nd* | *nd* | | *nd* |
| Creatinine (mg/dL)^e^ | **0.380*** | -0.258 | | 0.110 |

Supplemental Table 3. Correlations of oxygen gradient ektacytometry-derived biomarkers with laboratory parameters in the pediatric cohort.
‡p<0.0001, §p<0.001, †p<0.01, *p<0.,05.
^a^Total population of this cohort was 58 pediatric patients with SCD who were untreated or treated with hydroxyurea. Patients on (chronic) transfusion therapy (n=22) were excluded from this analysis.
^b^Data of 12 patients available, 46 patients had unknown data
^c^Data of 24 patients available, 34 patients had unknown data
^d^Data of 21 patients available, 37 patients had unknown data
^e^Data of 35 patients available, 23 patients had unknown data
HbF, fetal haemoglobin; HbS, haemoglobin S; Hb, haemoglobin; ARC, absolute reticulocyte count; MCV, mean corpuscular volume; MCHC, mean corpuscular haemoglobin concentration; LDH, lactate dehydrogenase; nd, not determined

**Supplemental Table 4. Effect of HU on oxygen gradient ektacytometry-derived biomarkers and laboratory parameters**

| **Effect of HU** | **HU treated cohort (n=15)** | | | |
| --- | --- | --- | --- | --- |
|  | **baseline** | **1 month** | **3 months** | **6 months** |
| **Oxygen gradient ektacytometry** | |  |  |  |
| PoS (mmHg) | 64.4 (35.1-87.6) | 61.6 (39.2-69.3 | 55.8 (33.5-60.4)**^§^** | 49.3 (37.4-56.7)**^‡^** |
| EI_min_ (EI) | 0.04 (0.00-0.23) | 0.08 (0.03-0.20) | 0.09 (0.05-0.31)**^†^** | 0.12 (0.07-0.22)**^‡^** |
| EI_max_ (EI) | 0.33 (0.22-0.56) | 0.36 (0.24-0.55) | 0.44 (0.31-0.55)***** | 0.45 (0.29-0.56)***** |
| **Laboratory parameter** |  |  |  |  |
| Dense Cells (%) | 8.3 (2.3-11.2) | 5.4 (5.0-9.9) | 6.3 (4.7-12.1) | 4.5 (3.2-12.1) |
| HbF (%) | 5.2 (0.9-28.5) | 7.5 (1.4-29.7) | 14.5 (4.1-33.7)**^‡^** | 17.5 (4.6-34.3)**^‡^** |
| HbS (%) | 82.0 (61.9-89.3) | 81.7 (60.7-88.7) | 73.3 (54.3-83.4)**^‡^** | 72.9 (56.3-83.3)**^‡^** |
| Hb (g/dL) | 9.0 (7.1-11.3) | 8.5 (6.9-13.0) | 9.6 (7.0-11.8) | 9.5 (7.3-11.5) |
| ARC (10^9^/L) | 348 (178-695) | 224 (98-399)**^†^** | 205 (50-346)**^§^** | 244 (48-445)**^‡^** |
| MCV (fl) | 79.1 (69.4-91.2) | 88.3 (75.1-116.0)***** | 98.8 (72.0-135.0)**^‡^** | 96.6 (77.2-130.0)**^‡^** |
| MCHC (g/dL) | 35.8 (34.2-40.0) | 35.8 (27.9-38.5) | 35.9 (32.2-38.8) | 35.6 (32.4-38.0) |
| RDW (%) | 23.0 (16.0-28.50) | 23.4 (16.8-28.3) | 19.6 (14.5-28.3)**^†^** | 18.5 (14.3-30.4)**^†^** |

Supplemental Table 4. Median values (range) of oxygen gradient ektacytometry-derived biomarkers and laboratory parameters before (baseline) and during hydroxyurea (HU) therapy (1, 3 and 6 months) of 15 patients with SCD (adult and pediatric). Baseline values are compared to 1, 3 or 6 months.
 ‡p<0.0001, §p<0.001, †p<0.01, *p<0.05.
HbF, fetal haemoglobin; HbS, haemoglobin S; Hb, haemoglobin; ARC, absolute reticulocyte count; MCV, mean corpuscular volume; MCHC, mean corpuscular haemoglobin concentration; RDW, red cell distribution width

**Supplemental Table 5. Effect of a single transfusion or chronic transfusion therapy on oxygen gradient ektacytometry-derived biomarkers and laboratory parameters.**

| **Effects of transfusion** | **Single transfusion** | | |  | **Chronic transfusion** | |  |
| --- | --- | --- | --- | --- | --- | --- | --- |
|  | **Before (single) transfusion** | | **After (single) transfusion** |  | **Before start of chronic transfusion** | **During chronic transfusion** |  |
| **Oxygen gradient ektacytometry** | | | **N=7** | |  | **N=21** | |
| PoS (mmHg)) | 47.3 (38.9-61.3) | | 42.0 (34.5-50.4)***** |  | 43.6 (30.9-64.0) | 37.4 (28.8-53.6)**^†^** |  |
| EI_min_ (EI) | 0.24 (0.04-0.36) | | 0.33 (0.17-0.39)***** |  | 0.08 (-0.02-0.36) | 0.21 (0.08-0.36)**^§^** |  |
| EI_max_ (EI) | 0.48 (0.27-0.58) | | 0.53 (0.37-0.58)***** |  | 0.47 (0.22-0.61) | 0.53 (0.39-0.60)**^†^** |  |
| **Laboratory parameter** |  | |  |  |  |  |  |
| Dense Cells (%) | 3.3 (1.9-10.5) | | 2.1 (1.9-7.2)***** |  | 5.4 (0.7-15.0) | 3.5 (1.5-7.3)**^†^** |  |
| HbF (%) | 7.7 (1.0-24.7) | | 4.9 (0.9-18.3)***** |  | 20.8 (10.8-41.2) | 19.4 (9.0-24.6) |  |
| HbS (%) | 47.6 (34.1-85.1) | | 28.8 (24.2-57.4)***** |  | *nd* | *nd* |  |
| Hb (g/dL) | 9.5 (7.1-10.7) | | 11.4 (9.7-11.8)***** |  | 8.5 (5.8-10.8) | 8.3 (7.4-11.6)***** |  |
| ARC (10^9^/L) | 228 (147-578) | | 189 (125-468)***** |  | 426 (205-836) | 405 (171-935) |  |
| MCV (fl) | 94.7 (83.7-111.0) | | 91.2 (86.0-103.0) |  | 95.2 (80.6-107.3) | 93.2 (79.3-107.4)***** |  |
| MCHC (g/dL) | 34.8 (34.3-38.4) | | 34.0 (33.5-36.6) |  | 32.9 (29.4-35.1) | 33.0 (29.1-34.7) |  |
| Platelet count (10^9^/L) | 321 (150-496) | | 260 (157-427) |  | 388 (233-946) | 419 (172-1465) |  |
| Neutrophils (10^9^/L) | 4.0 (1.4-14.3) | | 3.8 (1.5-11.7) |  | 4.0 (1.3-8.8) | 4.1 (1.8-6.6) |  |
| **Supplemental Table 5. Effect of a single transfusion or chronic transfusion therapy (pediatric cohort) on oxygen gradient ektacytometry-derived biomarkers and laboratory parameters.** Numbers represent median (range) or state otherwise. HbF, fetal haemoglobin; HbS, haemoglobin S; Hb, haemoglobin; ARC, absolute reticulocyte count; MCV, mean corpuscular volume; MCHC, mean corpuscular haemoglobin concentration; nd, not determined. ‡p<0.0001, §p<0.001, †p<0.01, *p<0.05. | | | | | | |  |

**
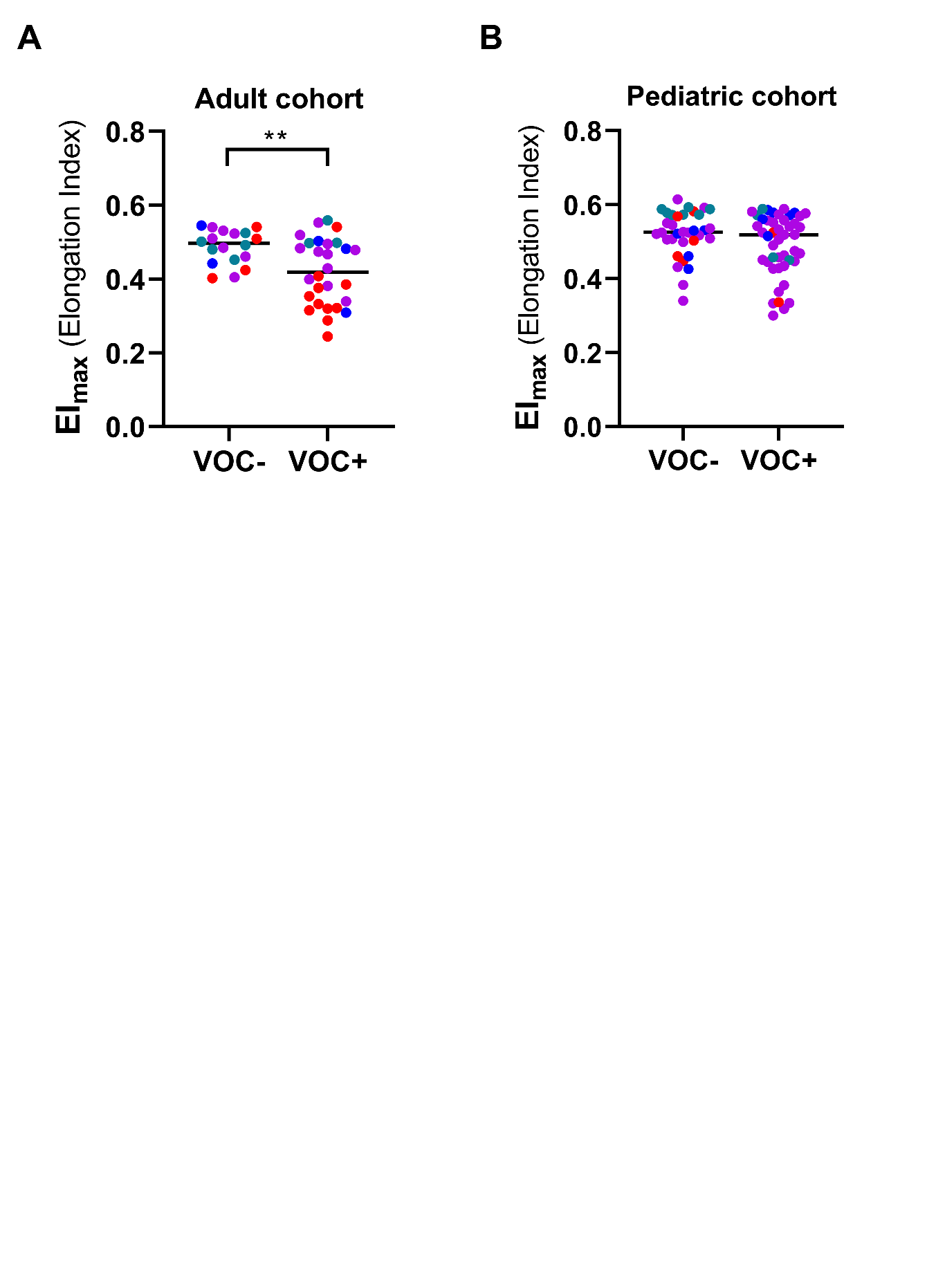
Supplemental Figure 1.**

Supplemental Figure 1. Oxygen gradient ektacytometry-derived biomarkers are associated with vaso-occlusive crisis. (A) Maximum deformability (EI_max_) is significantly lower in patients in the adult cohort with VOC compared to those without. Colors show different treatment regimens: untreated (red), HU treatment (purple), chronic transfusion (blue), HU and chronic transfusion (turquoise). (B) EI_max_ is not significantly different in patients in the pediatric cohort with VOC compared to those without. ***p<0.01*

**
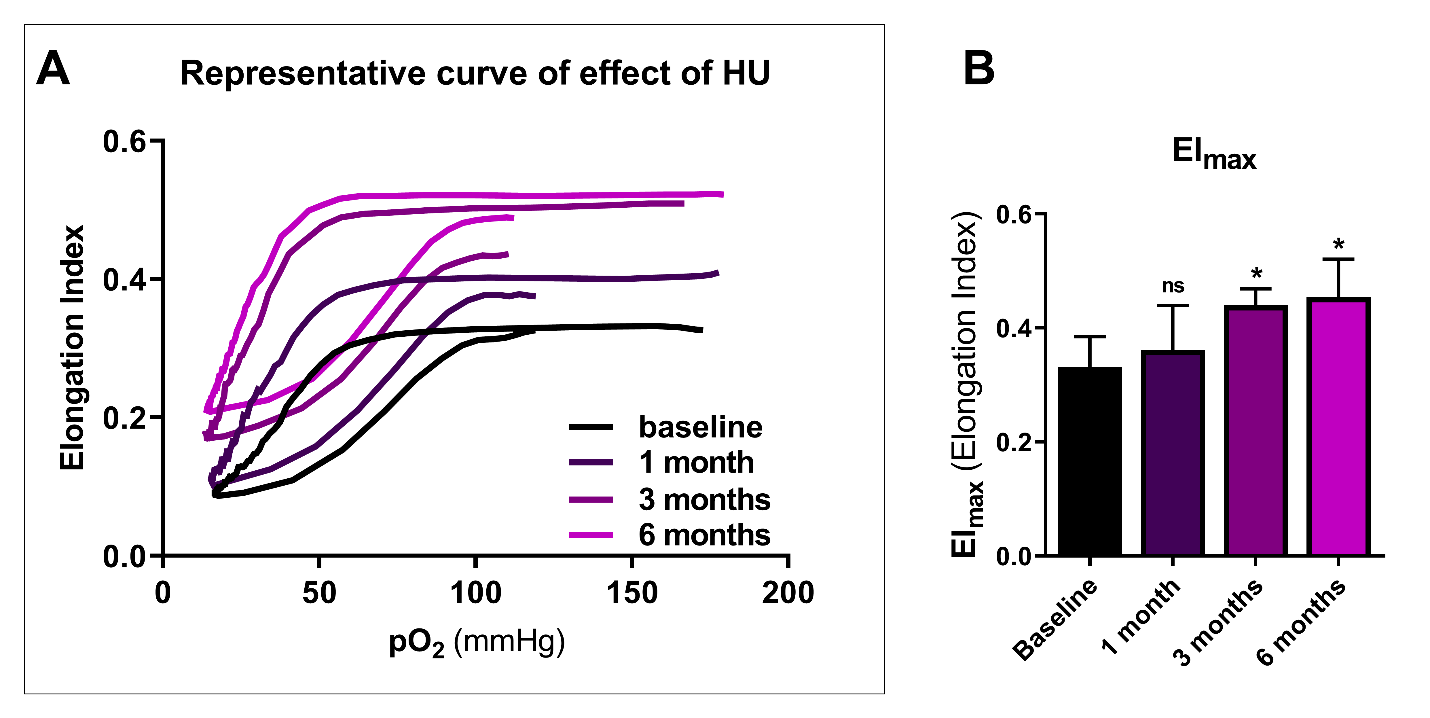
Supplemental Figure 2.**

**Supplemental Figure 2.** **Hydroxyurea has a measurable effect on oxygen gradient ektacytometry-derived biomarkers.** The effect of starting HU therapy was measured in 15 patients with SCD at baseline, and after 1,3 and 6 months of HU therapy. **(A)** Representative curve of a patient before and during hydroxyurea (HU) titration to maximum tolerated dose. **(B)** Median values of maximum deformability before deoxygenation (EI_max_) just before and during HU therapy. EI_max_ significantly increases after 3 and 6 months of HU compared to baseline values.
Error bars represent interquartile range. *****p<0.0001,* ****p<0*.*001, **p<0*.*01, *p<0*.*05*

**
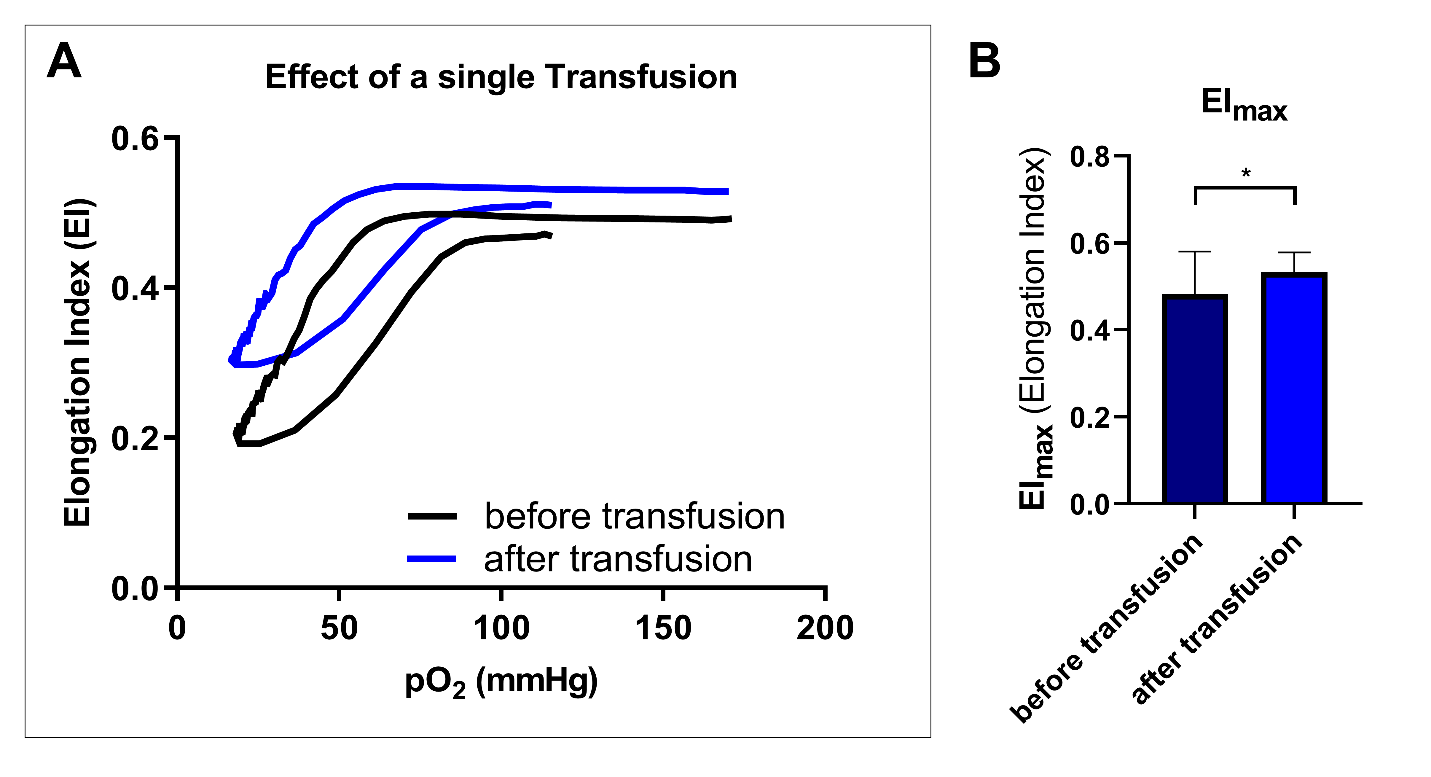
Supplemental Figure 3.**

Supplemental Figure 3. A single blood transfusion has a measurable effect on oxygen gradient ektacytometry-derived biomarkers. Seven patients with SCD were followed just before and after transfusion therapy. (A) Representative curve that highlights how blood rheology is improved by a blood transfusion. (B) Median values of EI_max_ are significantly decreased after transfusion, compared to before transfusion values. Error bars represent interquartile range.  **p<0*.*05*

**
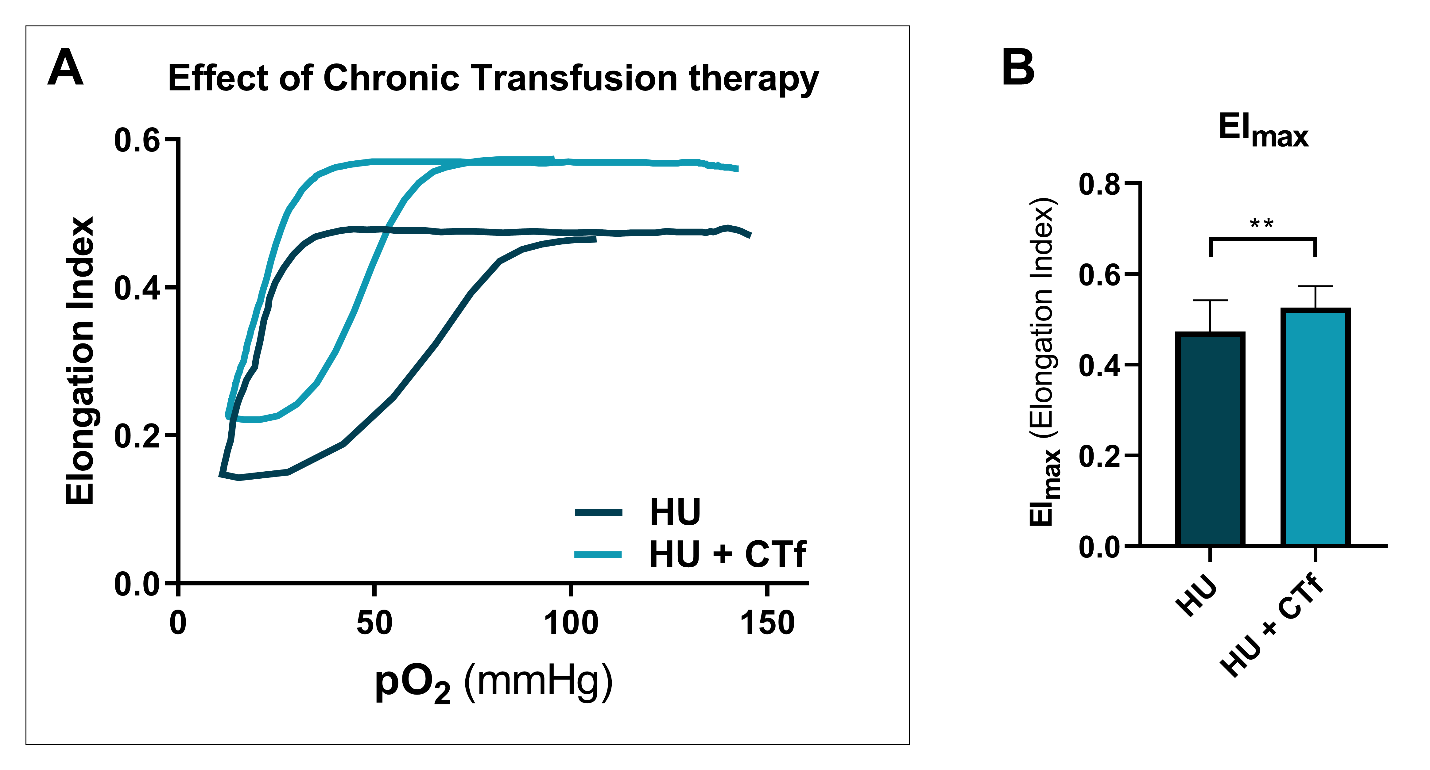
Supplemental Figure 4.**

Supplemental Figure 4. Chronic transfusion improves oxygen gradient ektacytometry-derived biomarkers in pediatric patients already treated with hydroxyurea therapy. Twenty-one patients with SCD were followed during HU and HU with CTf therapy. (A) Representative curve of a patient on hydroxyurea (HU) therapy before start of chronic transfusion therapy (CTf) and on CTf. (B) Median values of EI_max_ before and on CTf, that significantly decreases during CTf.
Error bars represent interquartile range*. **p<0*.*01*
